# The diagnosis of immune-related pancreatitis disguised as multifocal lesions on MRI by endoscopic ultrasound-guided fine-needle biopsy: A case report

**DOI:** 10.3389/fimmu.2022.933595

**Published:** 2022-09-13

**Authors:** Wen Shi, Bei Tan, Yuan Li, Liang Zhu, Yunlu Feng, Qingwei Jiang, Jiaming Qian

**Affiliations:** ^1^ Department of Gastroenterology, Peking Union Medical College Hospital, Chinese Academy of Medical Science and Peking Union Medical College, Beijing, China; ^2^ Department of Pathology, Peking Union Medical College Hospital, Chinese Academy of Medical Science and Peking Union Medical College, Beijing, China; ^3^ Department of Radiology, Peking Union Medical College Hospital, Chinese Academy of Medical Science and Peking Union Medical College, Beijing, China

**Keywords:** endoscopic ultrasound-guided fine needle biopsy, immune checkpoint inhibitor, pancreatitis, programmed death-1 antibody, immune-related adverse events

## Abstract

Immune checkpoint inhibitor (ICI)–related acute pancreatitis (irAP) is a rare, potentially life-threatening immune-related adverse event. Whereas CT and MRI remain first-line diagnostic imaging modalities, more patients are presenting with atypical irAP as ICI use increases. To appropriately manage these events, it is important to catalog these presentations and provide comprehensive clinical, radiological, and pathological descriptions to guide evidence-based practice. Here, we present the case of a 66-year-old man with advanced lung adenocarcinoma who, after the fifth course of toripalimab, developed epigastric discomfort and elevated serum amylase and lipase. irAP was suspected, but MRI revealed atypical, multifocal pancreatic lesions. To exclude metastases, an endoscopic ultrasound-guided fine-needle biopsy (EUS-FNB) was performed. EUS revealed a slightly swollen pancreas with heterogeneous echoic signals and scattered hyperechoic areas in the parenchyma without an obvious mass. Histopathological examination of the FNB revealed retention of the normal lobular pancreatic architecture with focal acinar atrophy associated with a CD8^+^ T lymphocyte-predominant infiltrate, further confirming the diagnosis of irAP. After starting glucocorticoids, his symptoms resolved, serum amylase and lipase rapidly decreased to normal, and the abnormal MRI features diminished. irAP can, therefore, present as multifocal lesions on MRI, and, when metastatic disease requires exclusion, EUS-FNB is an effective way to establish a definitive diagnosis. Refining the histopathological and immunopathological criteria for the diagnosis of irAP is now warranted.

## Introduction

Immunotherapy has dramatically revolutionized the therapeutic landscape for patients with cancer. Immune checkpoint inhibitors (ICIs) are now widely used in cancer management and include anti–programmed cell death protein 1 (PD-1) or anti–programmed cell death ligand 1 (PD-L1) inhibitors and cytotoxic T lymphocyte-associated antigen-4 (CTLA-4) inhibitors. However, it also introduces a novel class of toxicity, termed immune-related adverse events (irAEs). IrAEs range from self-limiting to life-threatening, leading to their temporary or permanent discontinuation and consequent life-threatening tumor progression. Therefore, the early diagnosis and effective treatment of irAE are clinically imperative to maximize the utility of immunotherapies.

ICI-related acute pancreatitis (irAP) is a rare yet serious irAE, with a reported overall incidence of 1% across all drug types (1). However, the incidence of irAP is growing as ICI use becomes more common. Classical irAP presents as diffuse peripancreatic enlargement, decreased enhancement, and fat stranding similar to autoimmune pancreatitis (AIP) on computed tomography (CT) and magnetic resonance imaging (MRI). However, these radiological features may be variable, even absent, creating diagnostic uncertainty as ICI use increases (2). Understanding the spectrum of radiological and pathological features is important to aid clinical decision-making in terms of further investigations and management. Here, we report a case of irAP presenting with multifocal lesions on MRI, with endoscopic ultrasound (EUS) revealing no evidence of a mass and histopathological examination of the fine-needle biopsy (FNB) confirming the diagnosis of irAE. Presenting this case provides an opportunity to improve our understanding of atypical irAP and its radiological and pathological manifestations in the ICI era.

## Case presentation

A 66-year-old man with left lung adenocarcinoma (cT1cN0M1b, stage IVb; *EGFR*, *ALK*, *BRAF* ^V600E^, *ROS1*, and *KRAS* mutation negative, PD-L1 unknown) had participated in a clinical trial studying standard-of-care pemetrexed and carboplatin with anti–PD-1 toripalimab therapy or placebo. While his disease initially stabilized, he later developed the progressive disease while on anti–PD-1/placebo monotherapy maintenance, and unblinding of the clinical trial indicated that he was in the placebo group. He was then started on toripalimab 240 mg every 3 weeks. After two courses of anti–PD-1 therapy, he achieved a partial response.

He developed epigastric discomfort after the fifth course of toripalimab, and physical examination revealed middle upper abdominal tenderness. The complete blood count and the liver/kidney/lipid panel were all within normal ranges. Serum amylase and lipase were raised [617 U/L (35–135 U/L) and 1,501 U/L (2–53 U/L), respectively]. High-sensitivity C-reactive protein and IgG4 were 2.77 mg/L (<3 mg/L) and 468 U/L (80–1,400 U/L), respectively. Peripheral blood T lymphocyte subset analysis showed only a CD8^+^HLA-DR^+^/CD8^+^ T-cell ratio increase to 56.2% (6.3%–23.8%), whereas the CD4^+^ T-cell, CD8^+^ T-cell, CD4^+^/CD8^+^, and CD8^+^HLA-DR^+^/CD8^+^ ratios were all within the normal range.

**Figure 1:**
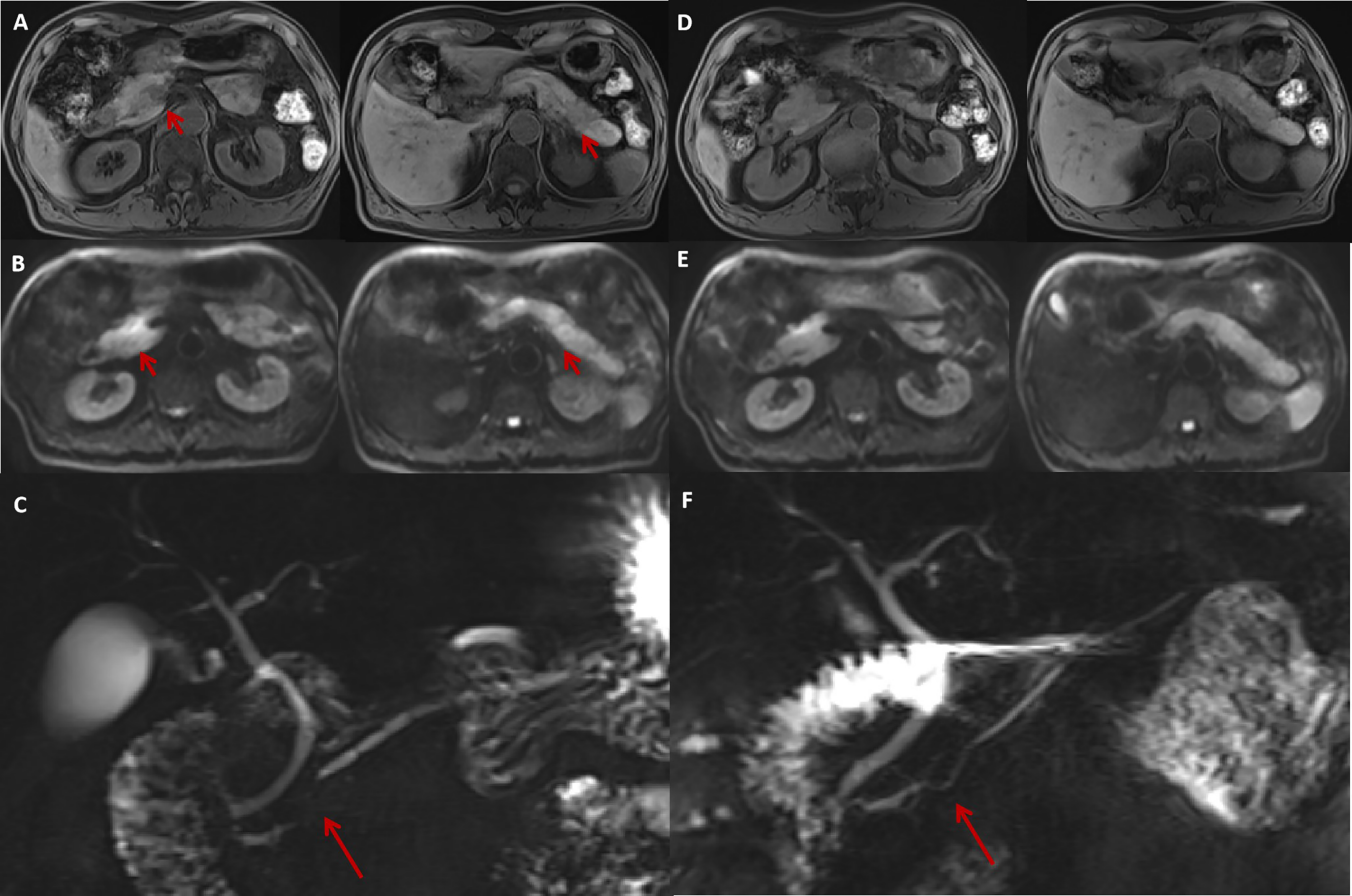
The MRI features of irAP and its improvement after treatment. The MRI at diagnosis revealed a slightly swollen pancreas with multiple nodular (**A**, arrow) and patchy abnormal signals with decreased intensity on T1WI sequences **(A)** and increased intensity on DWI (**B**, arrow) located in the pancreatic head and tail. MRCP showed segmental stenosis of the MPD in the pancreatic head (**C**, arrow). After 1 month of prednisolone treatment, the swelling reduced and the abnormal signals decreased on T1W1 **(D)** and DWI **(E)** sequences. The MPD stenosis also resolved (**F**, arrow). irAP, immune-related acute pancreatitis; MRI, magnetic resonance imaging; T1W1, T1-weighted image; DWI, diffusion-weighted imaging; MPD, main pancreatic duct; MRCP, magnetic resonance cholangiopancreatography.

The pancreatic morphology was unremarkable on abdominal contrast-enhanced CT (CECT), with homogeneous pancreatic parenchymal enhancement. MRI revealed multiple, nodular, patchy areas of abnormal signal intensity within the pancreas: slightly hypointense in T1-weighted images (T1WI), slightly hyperintense in T2-weighted images (T2WI), and significant diffusion restriction on diffusion-weighted imaging (DWI). There were two prominent lesions with blurred margins in the pancreatic head and tail accompanied by segmental stenosis of the main pancreatic duct (MPD). Furthermore, dynamic contrast-enhanced MRI showed heterogeneous enhancement of the pancreatic parenchyma, with relatively higher enhancement in the pancreatic body and tail in the arterial phase. The pancreatic head showed only slight hyper-enhancement, and no significant abnormal enhancement was observed in the delayed phase (**Figures 1A–C**).

**Figure 2:**
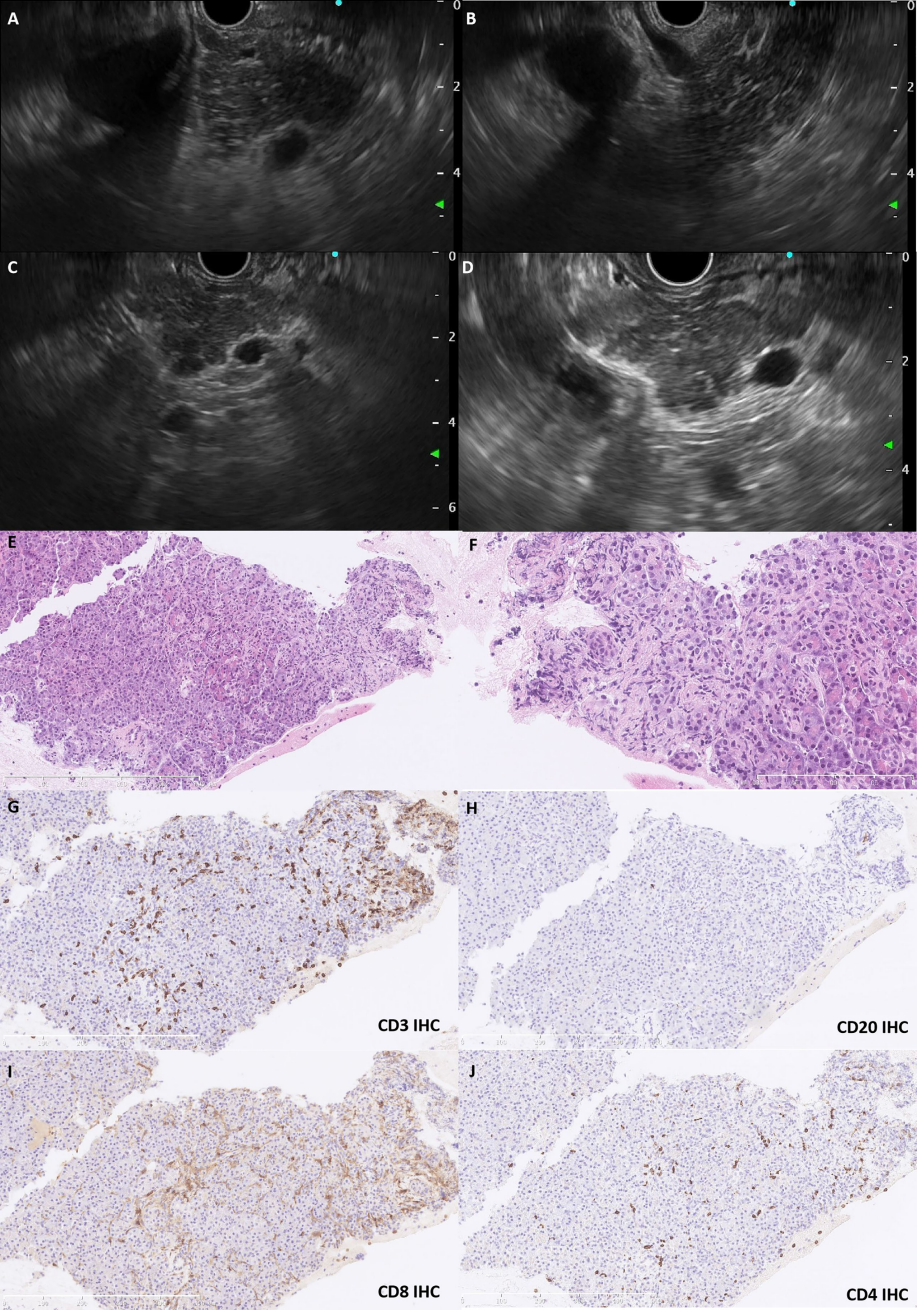
The EUS and histopathological features. EUS revealed a slightly swollen pancreas with heterogeneous echoic signals and scattered hyperechoic strips in the parenchyma **(A, B)**. The pancreatic duct was regular without dilatation, and the pancreatic tail was slightly enlarged without a mass lesion **(C, D)**. Pancreatic biopsy shows retention of the lobular architecture with focal atrophy of acini infiltrated with neutrophils and lymphocytes (**E**, HE staining,×150; **F**, HE staining, ×400). The lymphocytes were predominately T cells (**G**, CD3 IHC), although scattered B cells (**H**, CD20 IHC) were also present. CD8^+^ T cells (**I**, CD8 IHC) tended to dominate compared with CD4^+^ counterparts (**J**, CD4 IHC). EUS, endoscopic ultrasound; IHC, immunohistochemistry; HE, hematoxylin–eosin.

Because the lesions were multifocal, it was necessary to differentiate between metastases and irAP. Therefore, EUS-FNB was performed. On EUS, the pancreas was slightly swollen, and the echoic signals were slightly heterogeneous with scattered, striped hyperechoic areas in the parenchyma without obvious masses; the pancreatic tail was slightly enlarged (**Figures 2A–D**). An EUS-guided FNB of the pancreatic tail was taken with 19G needles (Expect, Boston Scientific, USA), fanning technique, and 5-ml suction by one needle pass. Histopathological examination of the needle core biopsy showed retention of the normal lobular architecture with focal acinar atrophy associated with a neutrophil and lymphocyte predominant infiltrate (**Figures 2E, F**) but no fibrosis. On immunohistochemical analysis, the lymphocytes were predominately CD8^+^ T cells (CD8^+^ > CD4^+^), although scattered B cells were seen (**Figures 2G–J**). There was no increase in IgG4^+^ plasma cells. The ductal epithelium was generally well preserved and was not associated with neutrophils. Immunohistochemical stains with antibodies targeting thyroid transcription factor 1 (TTF-1), IgG, and IgG4 were negative.

The patient was diagnosed with moderate irAP (grade 2), and he was treated with oral prednisolone 40 mg daily (0.7 mg/kg/day) with regular tapering of 5 mg every 2 weeks. His symptoms quickly settled, and his serum amylase and lipase had returned to normal 1 month later. Magnetic resonance cholangiopancreatography (MRCP) showed a decrease in the nodular and patchy abnormal signals, especially in the pancreatic body and tail (**Figures 1D, E**). The segmental stenosis of the MPD resolved with the consequent resolution of the distal MPD dilation (**Figure 1F**).

## Discussion and conclusion

irAP is more common with CTLA-4 inhibitors than PD-1 inhibitors, and the incidence is reported to be additive with a higher risk in patients with combined therapies (3). Although less common, our patient developed irAP with anti–PD-1 monotherapy toripalimab. IrAP-related imaging studies are scarce, with the most comprehensive series describing the CECT and MRI features in 25 irAP patients, typically diffuse (56%) or focal (44%) acute interstitial pancreatitis; no patient had a pancreatic tissue biopsy (4). There are even fewer reports of the EUS and pathological features of irAP. Here, we present a case of irAP presenting with multifocal lesions on MRI, with EUS-FNB helping to establish the definitive diagnosis and exclude primary or metastatic malignancy through histopathological analysis.

For patients with suspected irAP, National Comprehensive Cancer Network (NCCN) guidelines recommend abdominal CECT as the first-line examination. However, we and others have found that the CECT plays only a limited role in the diagnosis of irAP, with a sensitivity of only 17%. MRI is sensitive for detecting pancreatic abnormalities, especially when CECT is inconclusive (5). In our case, T1WI sequences showed slightly decreased signal intensity, whereas DWI demonstrated significant diffusion restriction resembling AIP and different from acute edematous pancreatitis. Our patient also had segmental MPD stenosis with secondary distal MPD dilatation, which is more suggestive of AIP and consistent with a previous case report (6).

The multifocal lesions seen on MRI in our case were unusual and initially caused diagnostic uncertainty. In patients with pre-existing malignancy, excluding metastases is essential, and EUS is a very useful diagnostic modality in this regard. Previous reports of EUS in irAP are extremely rare, with only two cases showing typical diffuse hypoechoic pancreatic enlargement with scattered hypoechoic areas and patchy and heterogeneous parenchyma, and another case revealing a hypoechoic mass in the pancreatic neck causing MPD stenosis (6–8). In our case, the pancreas was slightly swollen, and, in contrast to CECT and MRI, no obvious mass was seen on EUS. MRI is generally considered a sensitive method for detecting inflammatory changes in the pancreas, and heterogeneous inflammation can produce the appearance of multifocality on MRI. EUS is particularly sensitive for detecting masses, and even early-stage pancreatic carcinoma is more readily detected by EUS than CECT or MRI. Therefore, EUS is particularly useful for excluding space-occupying lesions and has the advantage that EUS-guided FNB can be used to obtain tissue to make a histopathological diagnosis, with 67% histologic capability and 93% accuracy (7). Because a false-negative result is possible through inadequate or unrepresentative tissue sampling, a final diagnosis might need to be confirmed by disease resolution with active management.

There are only two previous reports describing the cytopathological features of irAP, in which the inflammation was neutrophil-predominant (8, 9). Ours is the first case to describe the histopathological features of irAP, an analysis enhanced by immunophenotyping to establish the immune repertoire. We found that the pancreatic architecture was largely preserved, and neutrophils and lymphocytes infiltrated the pancreatic parenchyma but not the ductal epithelium. Type 2 AIP and other forms of chronic pancreatitis can have neutrophilic infiltrates within acinar units, but they often form microabscesses within duct lumina, which were absent in our case. In addition, the typical fibrosis seen in type 2 AIP was not present. Furthermore, both serum and tissue were negative for IgG4, which is a useful differentiation marker for type 1 AIP. An inflammatory infiltrate dominated by CD3+ T lymphocytes and a higher CD8+/CD4+ ratio can also support irAE and is consistent with the T-cell profile seen in a case of ICI-associated diabetes mellitus (10).

Our observations prompted us to speculate about the possible pathobiology of irAP. Anti–CTLA-4– and anti–PD-1–induced colitis have distinct immunological characteristics, with CD8+ and CD4+ T lymphocytes predominating in anti–PD-1– and anti–CTLA-4–induced colitis, respectively (11). In the case of pancreatic islet injury caused by combined anti–CTLA-4 and anti–PD-1 therapy, the low expression of PD-L1 indicated that β-cell injury was mainly associated with anti–PD-1 therapy, and the infiltrate was also CD8+ T cell–enriched, suggesting that CD8+ T-cell infiltrates are a more general feature of anti–PD-1 therapy. Immunophenotyping of T cells in pancreatic tissue biopsies may be useful for making the diagnosis of irAP, and further studies are now required to refine the diagnostic pathology criteria for irAP.

Interestingly, EUS also revealed scattered hyperechoic strips in the pancreatic parenchyma, which are a feature of early-stage chronic pancreatitis. Previous reports have shown that pancreatic atrophy developed in 11 of 25 (44%) irAP patients, and our previous study found that irAP can run a protracted course after glucocorticoid treatment (3, 5). IrAP has a tendency to become chronic and result in exocrine/endocrine insufficiency with a median 43% decrease in pancreatic volume (4, 10). Activated and increased CD8+ T cells damage pancreatic cells, further decreasing the number of pancreatic ductal and acinar cells to produce pancreatic atrophy (1). However, the imaging in the current case only suggested a sign of early pancreatic atrophy, and further studies are warranted to elucidate the pathophysiology and long-term progression of irAP (10).

This patient was diagnosed with moderate irAP; hence, the ICI was discontinued, and oral prednisolone 0.5–1 mg/kg/day was administered. Although Abu-Sbeih et al. found that intravenous fluids can potentially be beneficial to prevent long-term adverse outcomes from irAP (3), this was a retrospective study with a small sample size. Furthermore, 18 of 32 patients received only intravenous fluids without steroids suggesting mild disease and consistent with their excellent outcomes. In those patients with long-term adverse outcomes, 6 of 11 patients had neither intravenous fluids nor steroids, so the lack of active management may have played a role in these poor outcomes. Therefore, we gave glucocorticoid therapy without intravenous fluids to our patient with moderate irAP according to NCCN guidelines and our previous study (5), with good effect. Our patient still has a stable disease, but the resumption of an ICI can be considered if the tumor progresses.

In conclusion, irAP can present as multifocal lesions on MRI. In such cases, EUS is useful for distinguishing mass lesions from heterogeneous inflammation and to obtain tissue for histopathological examination. Histologically, irAP can show a relatively normal lobular pancreatic architecture with neutrophils, a CD8+ T lymphocyte predominance, and only scattered B cells without excess IgG4 plasma cells. Therefore, EUS-FNB is a helpful procedure for establishing the diagnosis of irAP, particularly when multifocal on traditional imaging, by excluding metastatic masses and establishing a definitive histopathological diagnosis.

## Data availability statement

The original contributions presented in the study are included in the article/Supplementary Material. Further inquiries can be directed to the corresponding authors.

## Ethics statement

Written informed consent was obtained from the individual(s) for the publication of any potentially identifiable images or data included in this article.

## Author contributions

WS: clinical information collection, draft of the manuscript, and case discussion. YL: histopathological information collection and analysis, histopathological figures selection, and case discussion. LZ: radiological information collection and analysis, radiological figures selection, and case discussion. YF: performing EUS-FNB and EUS figure selection and case discussion. QJ: guidance on EUS-FNB and EUS figure selection and case discussion. BT: clinical information collection, case discussion, refining the manuscript, and financial support. All authors contributed to the article and approved the submitted version.

## Funding

This study was supported by the Youth Program of National Natural Science Foundation of China [No.82000526], the General Program of Natural Science Foundation of Beijing Municipality [No.7192172], and the Health Research and Special Projects Grant of China [No.201002020, No.201502005].

## Conflict of interest

The authors declare that the research was conducted in the absence of any commercial or financial relationships that could be construed as a potential conflict of interest.

## Publisher’s note

All claims expressed in this article are solely those of the authors and do not necessarily represent those of their affiliated organizations, or those of the publisher, the editors and the reviewers. Any product that may be evaluated in this article, or claim that may be made by its manufacturer, is not guaranteed or endorsed by the publisher.
